# Deciding Under Uncertainty: The Case of Refractory Intracranial Hypertension

**DOI:** 10.3389/fneur.2020.00908

**Published:** 2020-08-20

**Authors:** Christos Lazaridis

**Affiliations:** ^1^Neurocritical Care Unit, Department of Neurology, University of Chicago Medical Center, Chicago, IL, United States; ^2^Section of Neurosurgery, Department of Surgery, University of Chicago Medical Center, Chicago, IL, United States

**Keywords:** traumatic brain injury, shared decision making, intracranial hypertension, intracranial pressure, decompressive craniectomy, expected utility

## Abstract

A challenging clinical conundrum arises in severe traumatic brain injury patients who develop intractable intracranial hypertension. For these patients, high morbidity interventions such as surgical decompression and barbiturate coma have to be considered against a backdrop of uncertain outcomes including prolonged states of disordered consciousness and severe disability. The clinical evidence available to guide shared decision-making is mainly limited to one randomized controlled trial, the RESCUEicp. However, since the publication of this trial significant controversy has been ongoing over the interpretation of the results. Is the mortality benefit from surgery merely a trade off for unacceptable long-term disability? How should treatment options, possible outcomes, and results from the trial be communicated to surrogates? How do we incorporate patient values into forming plans of care? The aim of this article is to sketch an approach based on insights from *Decision Theory*, and specifically *deciding under uncertainty*. The mainstream normative decision theory, Expected Utility (EU) theory, essentially says that, *in situ*ations of uncertainty, one should prefer the option with greatest expected desirability or value. The steps required to compute expected utilities include listing the possible outcomes of available interventions, assigning each outcome a utility ranking representing an individual patient's preferences, and a conditional probability given each intervention. This is a conceptual framework meant to supplement, and enhance shared decision making by assuring that patient values are elicited and incorporated, the possible range and nature of outcomes is discussed, and finally by attempting to connect best available means to patient-individualized ends.

Intracranial hypertension (IHT) has been associated with high mortality and poor outcomes after traumatic brain injury (TBI) (1, 2). Guidelines and experts advocate a “staircase” management approach wherein higher tiers involve therapies with higher propensity for adverse effects and a narrowing benefit to risk margin (3, 4). Clinical decision-making becomes particularly challenging for the 10–20% of patients refractory to first-line therapies (the requirement for stage-2 interventions increases the relative risk of death by 60%) (5). For these patients, rescue interventions include hypothermia, decompressive craniectomy (DC), and further metabolic suppression via barbiturate coma (BC) (6). The decision to offer a life-saving treatment for refractory IHT has to be balanced against great uncertainty in regards to functional outcomes and quality of survival; it is to be made in conjunction with surrogate decision makers via shared decision making (SDM) for establishing patient-specific goals (7, 8). A recent international consensus convened to provide direction on the use of DC following TBI; participants almost unanimously recommended that before contemplating DC for refractory IHT, providers should conduct frank discussions with surrogates regarding the risks, benefits, and potential alternatives (9). However, no insights were offered on how these discussions should be structured, how the alternatives should be presented, and how the potential outcomes should be understood. Furthermore, no guidance was furnished on how patient values are to be weighed in on choosing among alternatives.

This article attempts to offer a conceptual framework to assist clinicians think through the available choices, and to incorporate patient preferences into shared decision-making deliberations in the setting of refractory IHT after TBI. The plan of the article is as follows: the first section reviews clinical evidence with a focus on the Randomized Evaluation of Surgery with Craniectomy for Uncontrollable Elevation of Intracranial Pressure (RESCUEicp), an international, multicenter, parallel-group, superiority randomized clinical trial (RCT), that compared last-tier secondary DC with continued medical management including barbiturate coma (10). The second section offers a deliberation scheme based on principles of *Decision Theory*. The third section applies insights from decision theory to the conundrum of choosing among alternatives, and advising surrogates in the case of refractory IHT after TBI. At the end, limitations are discussed.

## Evidentiary Basis For Addressing Refractory IHT

RESCUEicp assessed the efficacy of DC after first- and second-tier therapies had failed to control refractory and sustained IHT in TBI patients. It is the only RCT that has aimed answering the clinical problem of *refractory* intracranial pressure (ICP) after TBI, and that allows a comparison of surgical decompression with most aggressive medical therapy including mild to moderate hypothermia and barbiturates [the preceding Decompressive Craniectomy -DECRA- trial is not analogous since it evaluated DC as an earlier measure to control ICP (11)]. RESCUEicp took over 10 years to complete, across 52 hospitals in 20 countries (although the majority of patients were enrolled in the UK). Such undertaking is unlikely to be repeated, and so this trial may serve as the only RCT-derived clinical guide (for the foreseeable future at least). The recruited cohort comprised TBI patients aged between 10 and 65 years, and ICP exceeding 25 mmHg for 1–12 h, despite first and second-tier measures for ICP control. The surgical treatment was a DC (either large unilateral frontotemporoparietal or bifrontal). The primary outcome measure was the Glasgow Outcome Scale-Extended (GOSE) at 6 months; 408 patients were recruited: 206 randomized to the surgical, and 202 to the medical group. At 6 months, the DC group had a significantly lower mortality rate compared to the medical group (26.9 vs. 49.9%). Surgery resulted into higher rates of vegetative state, lower severe and up*per se*vere disability, but the rates of moderate disability and good recovery were comparable. Favorable outcome was pre-specified, and dichotomized at up*per se*vere disability or better on the GOSE; 42.8% of surgical patients had a favorable outcome compared to 34.6% of medical patients (*p* = 0.12). At 12 months, 45.4% of surgical patients had a favorable outcome vs. 32.4% of medical patients (*p* = 0.01). It should also be noted that a large proportion (37%) of the medical treatment group did not achieve adequate ICP control and crossed-over to DC (the surgical crossover to BC was 9%).

Since the publication of the trial significant controversy has been ongoing over the interpretation of the results. Is this a positive trial for DC? Does the significant reduction in mortality merely translates to unacceptable long-term disability? How should these results be communicated to surrogates, and who should evaluate if an outcome is favorable or not? Critics of the trial objected that RESCUEicp did not follow the conventional definition of “favorable outcome” that is full independence at 6 months (12); under conventional dichotomization the proportion of patients with favorable outcomes would be similar for the two groups (27% at 6-months, and 32% surgical vs. 28.5% medical at 12-months). Others further argued that the point of comparison should be nothing less than disability-free survival, where there was no difference between the surgical and medical groups (9.8 vs. 8.4% at 12-months). According to such a criterion, surgery is not associated with any true long-term benefits; it only increases the number of patients in a vegetative state or suffering serious disability, and should therefore not be used (13). As a response, the investigators have in general defended DC under the premise that the unconventional dichotomization they chose was set a priori, and justified in view of these patients being in extremis. They have also argued that the final arbitrators of what is favorable or acceptable ought to be the patients, and their families (12, 14). The ensuing debate was highlighted early in the New England Journal of Medicine editorial for the trial (15). The authors of the editorial remarked that “quality of life is an individual determination, and it is important to engage surrogates in discussions that focus on patients' previously stated wishes and personal values”. They called for *the development of more refined clinical decision-making tools*, although no such account has been offered.

## Principles of Decision Theory: Deciding Under Uncertainty

Decision theory explores the reasoning underlying an agent's choices, whether this is a simple, trivial situation, or a far more involved, complex process as required in medical decision-making. A decision is said to be made *under ignorance* when no probabilistic information in terms of outcomes is available; the opposite prospect, where outcome probabilities are determined and known, refers to decision *under risk* (e.g., lotteries). In the real world, and in most medical decision-making, outcome probabilities are only partially determined, and with various degrees of confidence; this creates exemplary situations of *deciding under uncertainty* (16). Many lifesaving interventions produce a variety of outcomes, and no one can predict precisely where a particular patient will end up. Concurrently, it is extremely hard to anticipate how patients (and their caretakers) will evaluate and adapt to outcomes that leave them with various degrees of disability. We should also remain skeptical of disability-free or neurotypical peoples' capacity to predict how patients will evaluate these outcomes; this relates to the *disability paradox*, a significant underestimation (among able-bodied people) of actual quality of life associated with a certain disability (17). Relevantly, the disability paradox has been documented in surveys showing a disparity between what is considered a favorable outcome among healthy adults and patients treated with surgical decompression (18, 19).

In neurocritical care, we need a model for rationally guiding decisions applicable to situations that combine high degrees of multi-dimensional uncertainty over life-altering high-stake outcomes (20). The mainstream normative decision theory, Expected Utility (EU) theory, essentially says that, *in situ*ations of uncertainty, one should prefer the option with greatest expected desirability or value (21, 22). This article employs EU as a normative theory—that is, a theory of how people should make decisions (this differs from the approach in classical economics, where EU is often used as a descriptive or predictive theory). Roughly, we say that an agent “prefers” one option over another just in case, for the agent in question, the former is more desirable or choice-worthy than the latter. As one investigates rational preferences over prospects, the measurement of preference orderings will become important. The measures in question are known as *utility functions*. As long as the set of prospects is finite, any order can be represented by an ordinal utility function. The term U(O) represents the utility of a certain outcome—roughly, how valuable it is. Formally, U is a function that assigns a real number to each of the outcomes (the units associated with U are typically called *utiles*). The greater the utility, the more valuable the outcome. Assigning utilities to these options forces us to compare them. To say that X has greater utility than Y (for an agent) is simply to say that the agent prefers X to Y [to demonstrate, say that *u* is a utility function, it follows u(X) > u(Y)]. This is a depiction of how the preference relation can be represented as maximizing utility, since it favors the option with highest utility. The expected utility of an act is a weighted average of the utilities of each of its possible outcomes, where the utility of an outcome measures the extent to which that outcome is preferred, or preferable, to the alternatives. The utility of each outcome is further weighted according to the probability that the act will lead to that outcome. EU provides a way of ranking the acts according to how *choiceworthy* they are: the higher the expected utility, the better it is to choose the act.

Based on this framework, we can now rigorously define expected utility of possible medical interventions. The expected utility of an intervention i (in our scenario DC or BC) depends on two features of the problem: the value (how desirable it would be) of each outcome for the patient, and the probability of each outcome conditional on each intervention. Given these features, the expected utility of an i, EU(i), for different outcomes (O) can be conceptualized as a function of the product of the probability of a certain outcome P_i_(O) and *utility* of the outcome U(O). Following this, one could derive EU(DC) and EU(BC) for the different Glasgow Outcome Scale outcomes [this would lead to directly comparable expected utilities as for e.g., EU_GOSEx_(DC) vs. EU_GOSEx_(BC)]. For our purposes, will be employing intervention-contingent outcome probabilities as degrees of belief warranted by the evidence provided in RESCUEicp [see Table 3 in the original publication, reference (10)]. The *utility* of outcomes will be derived according to the values of the patient as elicited via SDM with surrogates. Even if it would be impossible to assign numeric values to these preferences, we can still use them as comparative modifiers in deriving expected utilities for the different interventions. The next section examines different patient-value preferences to exemplify how EU theory can rationalize SDM in the setting of refractory IHT.

## Decision Theory and Decompressive Craniectomy

The definition of shared decision making, as endorsed by the American College of Critical Care Medicine, is “a collaborative process that allows patients, or their surrogates, and clinicians to make health care decisions together, taking into account the best scientific evidence available, as well as the patient's values, goals, and preferences” (7). There are multiple nuances to this process, and merely “sharing” decision-making may not fulfill the above definition. Importantly, clinicians should be aware of cognitive biases (affecting clinicians and surrogates) that may operate at an unconscious level yet may influence behavior and potentially the care provided (8, 23). Cognitive biases and heuristics can affect SDM by distorting the understanding of the nature of a certain choice or decision and the foreseeable consequences (24, 25). A decision-theoretical model could shield SDM against biases, and enhance it by providing a method to rank available choices according to patient-specific values. What follows is an application of EU-guided decision-making to two refractory IHT scenarios as informed by different patient/surrogate preferences. For both scenarios, further action (in the form of DC or BC) is to be understood as primarily life-preserving in the sense that without it there is very high likelihood that the patient will die. Surrogates ought to be also informed that although these interventions confer a significantly higher chance for survival, possible outcomes are highly uncertain and include the potential for prolonged, and severe, physical and neurocognitive disabilities. The first scenario is one where the patient would foremost opt for the intervention associated with the highest chances for preserving life. In the second scenario, the patient would foremost opt for the intervention associated with the highest chances for functional independence. Preferences focusing on saving life, or on functional independence are the most common considerations that come up in neurocritical care family conversations. In emergency situations involving life-threatening neurologic illness, people want to know what can be done to preserve life, and also if a certain intervention or treatment paradigm can restore as much as possible of premorbid function. A clinical caveat to be recalled is that age (RESCUEicp had an age limit of 65) and comorbidities may affect eligibility, risk, and outcomes of anti-IHT treatments.

### Preserve Life

Formally, in terms of EU theory, the utilities of different outcomes can be represented by U_GOSE2−8_ > U_GOSE1_. Outcomes from RESCUEicp (understood as probability distributions contingent to each intervention) show that DC confers a 22% absolute risk reduction for mortality, and a number needed to treat of 5; it follows that EU_GOSE2−8_(DC) > EU_GOSE2−8_(BC). For this preference ranking, decompressive craniectomy is the rational choice to make in line with the value to foremost preserve life.

### Functional Independence

Here, the utilities of different outcomes is represented by U_GOSE5−8_ > U_GOSE1−4_. By using the outcomes distribution, it appears that EU_GOSE5−8_ (DC) ≈ EU_GOSE5−8_ (BC). The two interventions have similar chances leading to an outcome of functional independence. It would be important though to further understand if the patient would consider a life of functional dependence as a life not worth living. Such a stance would favor the intervention associated with higher mortality but less vegetative state and severe disability, meaning BC (DC increases the absolute risks for vegetative state by 6%, and severe disability by 15%). A difficult dilemma would remain in the case of refractory IHT despite BC (recall the 37% cross over). RESCUEicp results are based on intention-to-treat analysis; it would be very helpful to have specific empirical data on the outcomes of patients who crossed-over to surgery. The argument has been made that if a substantial portion of these patients went on to make favorable long-term recoveries that would be grounds to argue in favor of DC. Otherwise, support for surgery would be seriously called into question (26).

### Accepting Upper Severe Disability

**Figure 1:**
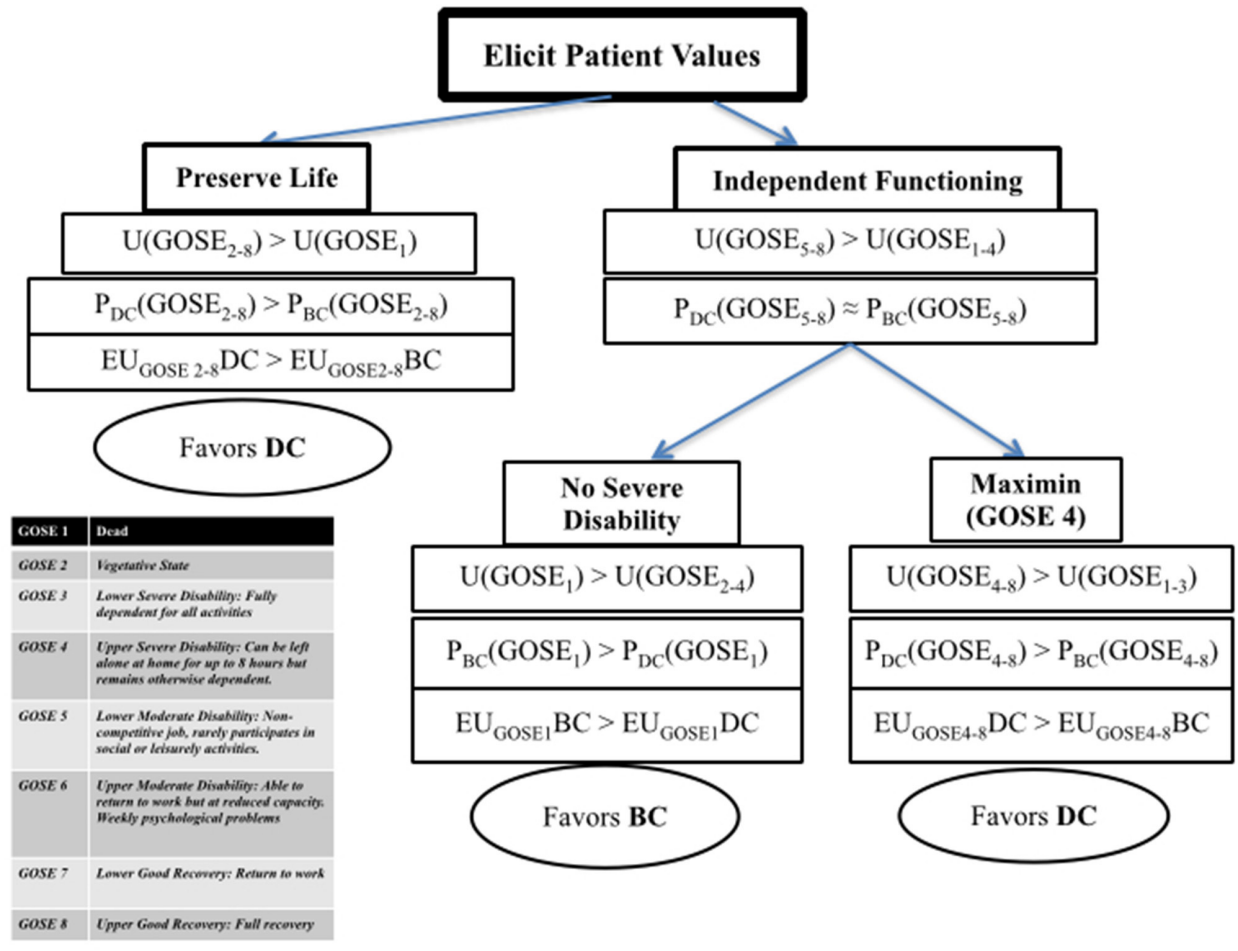
Expected utility guided decision making for refractory intracranial hypertension. U, utility of an outcome; GOSE, Glasgow Outcome Scale-Extended; P, probability of an outcome; EU, expected utility (function of the product U and P); DC, decompressive craniectomy; BC, barbiturate coma.

The verdict would again change for another patient who would minimally accept up*per se*vere disability (GOSE 4). This is somebody who would value their independent ability to spend time at home, even if this would entail total dependency on others for outside activities. This approach introduces a *maximin rule* (“maximize the minimum” regret or loss to well-being) where via SDM we would attempt eliciting the minimally acceptable vs. death outcome for a given patient. If this threshold is set at up*per se*vere disability, that would favor DC as the recommended strategy (Figure 1 offers a depiction of the above process and conclusions; Box 1 provides a case example to illustrate the process).

Box 1 **Case Example.**

A 57-year-old man was admitted with severe diffuse TBI after a car accident, and developed refractory ICP despite first and second tier measures. An urgent family meeting was held. His surrogates described the patient as someone who lived alone mostly spending his time reading at home, who would also enjoy visits from friends and family, and who would also seek opportunities to travel. He had consistently said that he would prefer to be dead than vegetative, but no advance directive existed. As the next step in management, the medical team discussed the options of either a decompressive craniectomy or barbiturate coma for control of ICP; the family was informed about the results of a recent clinical trial showing that for every 100 patients treated with DC rather than barbiturates, there were 22 more survivors; of these, six were in a vegetative state, and the other 16 were dependent on daily support for combinations of cognitive and physical disabilities. Family members disagreed about the best course of action.

In applying the flowchart from Figure 1 for this patient, one would have to follow the “independent functioning” path since this patient's previously expressed wishes were against preserving life at all costs as he considered survival in a vegetative state as worse than death. Here, the family was further asked what possible level of dependency the patient would potentially deem acceptable, if any. The more direct question to the family was if partial dependency with the ability to have unsupervised hours at home would be acceptable. The family was also informed that the patient could potentially achieve further functional improvement, however this was highly uncertain at the time being. Surrogates agreed that a functional state of home-independency, even if dependent for outside activities, would not be against the patient's wishes and interests. Such a stance would lead to the “Maximin” side of the chart, recommending a DC as the action maximizing expected utility in this scenario, for this patient (this is only an example illustrating how one could consult an EU-inspired approach to decision-making; it is meant to complement a further nuanced approach that would include patient-specific features such as neuroimaging and multimodality monitoring data, other organ-system function and co-morbidities, as well as family and social factors that could impact long term care).

## Limitations

The presented model purports to offer a “formula” for guiding decision-making based on ranking of outcomes according to patient values, together with probabilistic information of alternative interventions associated with these outcomes. An objection is that it leaves out potentially relevant considerations that should weigh in, such as social utility, allocation of scarce resources, and cost-benefit analyses (27, 28). These are indeed important variables, however the multidimensional uncertainty (empirical and ethical) that surrounds the care of acutely brain-injured patients complicates significantly any effort to incorporate such considerations early on. Nevertheless, there is little doubt that SDM would significantly benefit from data on identifying patient and injury phenotypes that would recommend them for one treatment approach over another, and reliable predictors of longer-term outcomes. Saliently, these outcomes should be evaluated from the perspectives of patients, their families and caretakers (29). RESCUEicp has provided 6 and 12-month outcomes; ongoing planned analysis is anticipated to provide 24-month outcomes. Natural history of recovery from brain injury can be significantly longer than what is usually recorded and reported. Contemporary series with extended follow-ups provide encouraging data in terms of recovery potential beyond the first year from injury, and show that GOSE categories as reported from trials are not necessarily static end-states (30, 31). In terms of DC, surrogates should be also informed about the need of additional future surgery such as cranioplasty, which carries both promise in terms of neurologic function improvement, and concern due to its own moribidity and complications (32). Finally, any use of outcome probabilities directly from RESCUEicp, as priors, should take into account the particularities of the trial, some mentioned earlier; in addition, one should consider the technical fact that most decompressions in the trial were bifrontal vs. unilateral.

## Conclusion

A most challenging clinical conundrum arises in severe TBI patients who develop life-threatening intractable intracranial hypertension. For these patients, last tier, high morbidity interventions, such as surgical decompression or pharmacologic coma, have to be considered against a backdrop of uncertain outcomes including prolonged states of disordered consciousness and severe disability. The clinical evidence basis available to guide shared decision-making is limited. Concurrently, there are no decision aids that could assist in rationally navigating available options, describing the nature and range of outcomes to surrogates, and incorporating patients' values into goals of care. The aim of this article has been to sketch such an approach employing insights from Expected Utility theory. The steps required to compute expected utilities include listing the possible outcomes of available interventions, assigning each outcome a utility ranking representing an individual patient's preferences, and a conditional probability given each intervention. This is not an algorithmic procedure meant to substitute for involved and nuanced shared-decision making, nor it promises to solve difficult real-world clinical dilemmas by a simplistic calculus like process. It is meant to supplement, and enhance SDM by assuring that patient values are elicited and incorporated, the possible range and nature of outcomes is discussed, and finally by attempting to connect best available means to patient-individualized ends.

## Data Availability Statement

The original contributions presented in the study are included in the article/supplementary material, further inquiries can be directed to the corresponding author/s.

## Author Contributions

CL conceived and drafted the manuscript.

## Conflict of Interest

The author declares that the research was conducted in the absence of any commercial or financial relationships that could be construed as a potential conflict of interest.
